# Genetic insights into underground responses to *Fusarium graminearum* infection in wheat

**DOI:** 10.1038/s41598-018-31544-w

**Published:** 2018-09-03

**Authors:** Kai P. Voss-Fels, Lunwen Qian, Iulian Gabur, Christian Obermeier, Lee T. Hickey, Christian R. Werner, Stefan Kontowski, Matthias Frisch, Wolfgang Friedt, Rod J. Snowdon, Sven Gottwald

**Affiliations:** 1 Department of Plant Breeding, IFZ Research Centre for Biosystems, Land Use and Nutrition, Justus Liebig University, Heinrich-Buff-Ring 26-32, 35392 Giessen, Germany; 2 Queensland Alliance for Agriculture and Food Innovation, The University of Queensland, St Lucia, QLD 4072 Australia; 3 Collaborative Innovation Center of Grain and Oil Crops in South China, Hunan Agricultural University, Changsha, 410128 P.R. China; 4 W. von Borries-Eckendorf GmbH & Co. KG, Hovedisser Str. 92, 33818 Leopoldshöhe, Germany; 5 Institute for Agronomy and Plant Breeding II, IFZ Research Centre for Biosystems, Land Use and Nutrition, Justus Liebig University, Heinrich-Buff-Ring 26-32, 35392 Giessen, Germany

## Abstract

The ongoing global intensification of wheat production will likely be accompanied by a rising pressure of *Fusarium* diseases. While utmost attention was given to *Fusarium* head blight (FHB) belowground plant infections of the pathogen have largely been ignored. The current knowledge about the impact of soil borne *Fusarium* infection on plant performance and the underlying genetic mechanisms for resistance remain very limited. Here, we present the first large-scale investigation of *Fusarium* root rot (FRR) resistance using a diverse panel of 215 international wheat lines. We obtained data for a total of 21 resistance-related traits, including large-scale Real-time PCR experiments to quantify fungal spread. Association mapping and subsequent haplotype analyses discovered a number of highly conserved genomic regions associated with resistance, and revealed a significant effect of allele stacking on the stembase discoloration. Resistance alleles were accumulated in European winter wheat germplasm, implying indirect prior selection for improved FRR resistance in elite breeding programs. Our results give first insights into the genetic basis of FRR resistance in wheat and demonstrate how molecular parameters can successfully be explored in genomic prediction. Ongoing work will help to further improve our understanding of the complex interactions of genetic factors influencing FRR resistance.

## Introduction

Global wheat production must be significantly increased in coming decades to meet future demands of the rapidly growing world population^1^. Increased production is accompanied by a rising pressure of various wheat pathogens, particularly fungi that can cause severe diseases such as rusts, powdery mildew or *Septoria*^2^. The forecasted climatic changes, such as increasing severe heat or drought events^3^ further exacerbate this danger by increasing the potential for emergence of new or presently minor pathogens^4^. *Fusarium* diseases including head blight (FHB), crown (FCR) and root rot (FRR) are of particular importance due to inherent plant protection management challenges and a lack of major resistances in modern elite varieties, and the highly complex genetic control of available resistances^5,6^. Resistance studies for FHB have identified over 200 significant quantitative trait loci (QTL) covering all 21 wheat chromosomes^7^, yet comparatively few studies have investigated *Fusarium* diseases in the context of root infections. However, various *Fusarium* ssp. can cause root rot, crown rot or seedling blight in all major wheat growing regions worldwide. Yield losses as high as 25% have been reported in Australia^8^ and 35–61% in North America^9^, respectively. Importantly, there is evidence for fundamental differences between wheat plant resistance responses against above-ground and below-ground plant infections with *Fusarium*. For example, identified resistance loci for FHB and FCR did not co-locate in a QTL mapping study by Li *et al*.^10^, whereas there were no major FHB resistance QTL detected in a mapping population carrying resistance to FCR^11^. This highlights the limited value of data accumulated from FHB research for resistance improvement of FCR and FRR. Growing resistant varieties is recognized as being a major solution for minimizing damage by below-ground *Fusarium* diseases. Reliable diagnostic markers could greatly support the improvement of durable resistances in modern varieties^12^, e.g. via gene pyramiding, yet knowledge about the underlying genetic factors is limited. While published genetic studies on FRR and FCR resistance have predominantly focused on *F*. *culmorum* or *F*. *pseudograminearum* using bi-parental QTL identification approaches, resistances effective in a bi-parental mapping population are often ineffective when transferred to different genetic backgrounds^13–15^. On the other hand, genome-wide association analysis using genetically diverse, unrelated populations could provide much more insight into diversity that has broader usefulness for breeding^16^. Very little is known about the effects on plant performance after below-ground infection with *F*. *graminearum* (Fg), a major causal agent for FHB^17^ that is also capable of infecting roots and stem tissue^18–20^. Large-scale QTL or GWAS analysis for root susceptibility to Fg is extremely challenging due to the inaccessibility of the roots for phenotypic evaluations in large populations. Consequently, the level and the genetic architecture of resistance against below-ground infections with Fg in global wheat germplasm remain vastly undescribed.

Here, we present the first large-scale genome-wide association study (GWAS) of seedling resistance towards FRR caused by Fg. We phenotyped a diverse collection of 215 international hexaploid wheat lines in a comprehensive greenhouse screen after root infection with fungal spores. In addition to the assessment of disease symptoms and biomass reduction of different plant parts, we quantified fungal spread in root and shoot tissues by RealTime-PCR as an assay for the relative quantity of pathogen DNA in comparison to the plant DNA in root tissues. Using genome-wide single-nucleotide-polymorphism (SNP) data for haplotype analyses we identified conserved chromosome regions associated with resistance and provide first insights into the complex genetic architecture of FRR seedling resistance in wheat. Our findings demonstrate the potential to improve quantitative resistance via haplotype stacking and provide a valuable basis for further molecular validations and genomics-assisted breeding to improve FRR resistance in future wheat varieties.

## Results

### Fungal root infections impacting seedling traits can be effectively assayed by RealTime-PCR

After infecting seedling roots of 215 wheat accession (Tables S1–2) with Fg spores in a glasshouse experiment, we assayed root dry mass (RDM), leaf dry mass (LDM), root length (RL), shoot length (SL) and the root-to-shoot ratio (R/S) along with a stem-base discoloration score for each accession. Phenotype values for all of these traits were significantly lower in inoculated plants compared to the control (p < 0.05), with the strongest effect on RDM (Figures S1, S2). Discoloration score of the stem-base ranged from 2 to 4.5, with half of the 215 analyzed accessions exhibiting a score of 3.3 or higher. On the roots, however, disease symptoms were not evident. The proportion of fungal DNA obtained by qPCR ranged from 0 to 0.0710 ng per 15 ng total DNA in the stem-base and from 0 to 0.0267 ng per 15 ng total DNA in root tissues, respectively. Fold-change (fc) values for the fungus-specific DNA fragment 16N (Fg DNA) relative to the wheat-specific housekeeping gene *Ubiquitin* ranged from 0 to 0.0189 in the stem-base and from −0.0012 to 0.0194 in roots. The measures for absolute fungal DNA relative to the biomass of infected plant roots (Fg_DNA_root_rel) ranged from 0 to 1.0236 (Table 1, Figure S3). As expected a significant positive correlation exists between the discoloration score at the stem base and the fungal DNA concentration in the roots (Figure S4). In contrast, no significant correlation exists between the discoloration score at the stem base and the fungal DNA concentration in the stem, suggesting that infection in the roots triggers symptom expression at the stem base. Interestingly, a higher biomass of roots (and stems) in control as well as in inoculated treatments is negatively correlated with fungal DNA concentrations in roots. Broad-sense heritability ranged from 0.27 for Fg_DNA_root_rel to 0.88 for SL_c and was generally was very similar under controlled and infected conditions. The only exception was the root length (RL), for which the heritability was only 0.46 under controlled conditions but 0.62 under infection, respectively.

**Table 1:** Summary of phenotype data for the Fusarium root infection experiments.

Treatment	Parameter	Unit	#Lines tested	Mean	Median	SD	SE	Var.	Min.	Max.	H^b^	V_g_^a^	sed ^b^	V_d_^c^
Control	LDM_c	[g/plant]	215	0.36	0.36	0.06	0.00	0.00	0.20	0.52	0.77	0.0025	0.039	0.001
	RDM_c	[g/plant]	215	0.09	0.09	0.04	0.00	0.00	0.03	0.32	0.76	0.0008	0.022	0.0005
	SL_c	[cm]	215	48.63	47.89	6.29	0.43	39.55	32.92	69.76	0.88	33.706	2.983	8.901
	RL_c	[cm]	215	25.01	25.19	3.58	0.24	12.85	15.70	34.29	0.46	3.824	2.967	8.804
	RS_c		215	0.25	0.24	0.09	0.01	0.01	0.07	0.75	0.69	0.0033	0.054	0.003
Infected	LDM_i	[g/plant]	215	0.31	0.32	0.07	0.00	0.00	0.12	0.60	0.81	0.0031	0.038	0.001
	RDM_i	[g/plant]	215	0.07	0.07	0.03	0.00	0.00	0.02	0.21	0.72	0.0003	0.015	0.0002
	SL_i	[cm]	215	47.29	46.93	6.05	0.41	36.63	29.95	70.12	0.81	30.382	3.744	14.017
	RL_i	[cm]	215	23.47	23.42	3.52	0.24	12.36	11.84	32.82	0.62	7.272	3.016	9.099
	RS_i		215	0.23	0.23	0.05	0.00	0.00	0.10	0.49	0.64	0.0013	0.039	0.002
Relative values	LDM_r		215	0.86	0.85	0.14	0.01	0.02	0.40	1.35	0.44	0.0083	0.146	0.021
	RDM_r		215	0.85	0.81	0.29	0.02	0.08	0.20	2.52	0.62	0.0397	0.219	0.048
	SL_r		215	0.98	0.98	0.07	0.01	0.01	0.72	1.18	0.55	0.0033	0.073	0.005
	RL_r		215	0.95	0.93	0.17	0.01	0.03	0.60	1.57	0.56	0.0149	0.154	0.024
	RS_r		215	0.99	0.96	0.32	0.02	0.10	0.37	3.24	0.62	0.0378	0.216	0.046
RT-PCR	Fg_DNA_stem_abs	[ng Fg DNA per 15 ng total DNA]	191	0.0124	0.0107	0.0122	0.0009	0.0001	0.0000	0.0710	0.33	0.00009	0.019	0.0004
	Fg_DNA_stem_fc	[E^−ΔCt^]	190	0.0049	0.0037	0.0040	0.0003	0.0000	0.0000	0.0189	0.49	0.00005	0.010	0.0001
	Fg_DNA_root_abs	[ng Fg DNA per 15 ng total DNA]	213	0.0083	0.0076	0.0055	0.0004	0.0000	0.0000	0.0267	0.40	0.00001	0.005	0.00003
	Fg_DNA_root_fc	[E^−ΔCt^]	213	0.0043	0.0035	0.0037	0.0003	0.0000	−0.0012	0.0194	0.60	0.00001	0.003	0.00001
	Fg_DNA_root_rel		213	0.1421	0.1056	0.1399	0.0096	0.0196	0.0000	1.0236	0.27	0.0044	0.155	0.024
	Discoloration score		215	3.265	3.319	0.515	0.035	0.266	1.976	4.451	0.57	0.138	0.460	0.212

LDM = Leaf dry mass, RDM = Root dry mass, SL = Shoot length, RL = Root length, RS = Root-to-shoot ratio, Fg = Fusarium graminearum.

SE = Standard error, SD = Standard deviation.

H^2^ = Heritability.

^a^Genotypic variance; ^b^Standard error between the difference of two line means; ^c^Average variance between two line means.

### Total number of resistance-associated haplotype alleles associates with discoloration score

**Figure 1:**
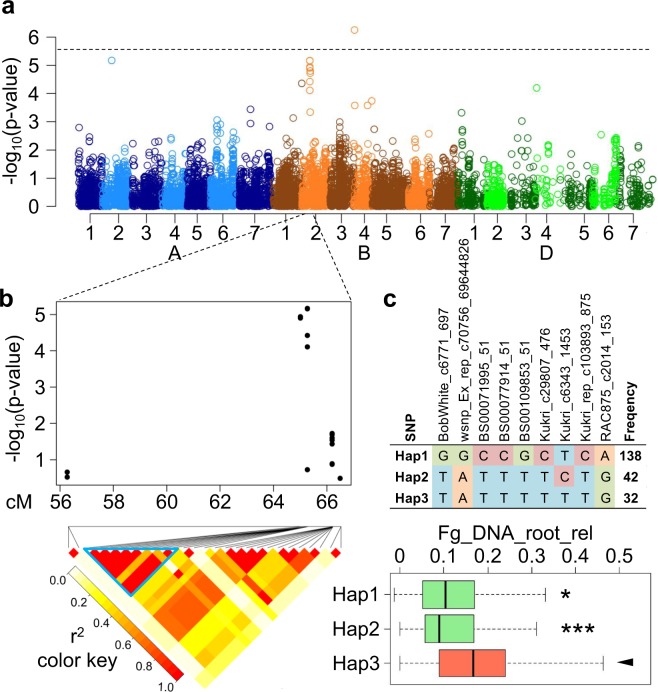
Resistance haplotype construction based on genome-wide association mapping (GWAS) for one of six identified haplotypes. (**a**) Manhattan plot for the trait “fungal DNA relative to the biomass of infected plant roots” (Fg_DNA_root_rel). Each colored dot represents the −log_10_(p-value) of a marker-trait association. The dashed line represents the Bonforroni threshold of −log_10_(p-value) = 5.57. (**b**) Close-up of the Manhattan plot in the 2B region. Heat map shows pairwise linkage disequilibrium values measured as r^2^ between single nucleotide polymorphism (SNP) markers. Blue triangle highlights the block of SNPs that was considered as one haplotype. (**c**) Haplotype block based on nine SNP markers. Three different haplotype variants (Hap1–Hap3) are present at different frequencies in the analyzed population. Boxplots indicate the phenotype values corresponding to the three different haplotype groups. Hap1 and Hap2 were associated with a significantly (t-test) lower relative fungal DNA in the roots and were therefore considered as “resistance haplotype alleles”.

**Figure 2:**
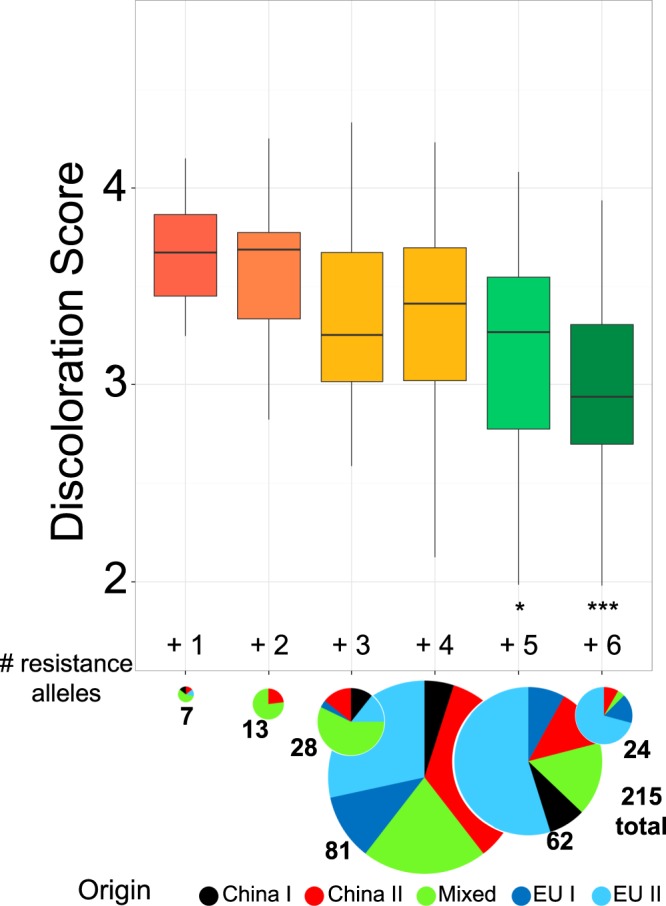
Comparison of discoloration score and number of resistance haplotype alleles possessed by the wheat lines among the diversity panel. Six previously defined haplotypes were considered. Pie charts indicate the origin of the genotypes in each of the six groups and the numbers represent the total number of genotypes in each group. A t-test was used to compare the discoloration score between group 1 and 6 (p-values: − 0.1, *0.05 **0.01 ***0.001).

Data from the Wheat 90 K Illumina Infinium array^21^ was used for GWAS and local LD analysis, resulting in a total of 95 putative marker-trait associations exceeding a threshold of −log_10_(p-value) > 3 (Table S3). Only one marker-trait association, for the trait Fg_DNA_root_rel, showed a p-value above the Bonferroni threshold (−log_10_(p-value) >5.57, Fig. 1), whereas p-values were distinctly lower for all other investigated traits. Given the complex nature of the trait, however, it can be expected that a number of the putative associations below the Bonferroni threshold actually represent true genetic effects of fungal infection. We therefore tested whether accumulation of allelic haplotypes showing putative associations to FRR resistance impart a significant phenotypic effect. To do this, all accessions in the diversity panel were assigned to groups depending on the absolute number of putative resistance–associated haplotypes they carry. This resulted in six different groups, ranging from seven lines which carried only one resistance–associated haplotype up to 24 lines that possessed all six putative resistance-associated haplotypes (Fig. 2). Comparison of discoloration scores among these groups revealed a very clear trend. While lines that carried only one of the six identified resistance-associated alleles showed a comparatively high discoloration score after fungal infection, the discoloration score decreased continuously with an increasing number of resistance–associated alleles. In total, 86 accessions were detected that carry a total of five or six resistance-associated alleles. More than 50% of the accessions with six resistance-associated alleles had discoloration score levels below three (Fig. 2). Interestingly, the majority of accessions with at least five resistance alleles had a European genetic background, whereas only a small number originated from China.

### Genomic prediction of resistance against seedling infection

**Figure 3:**
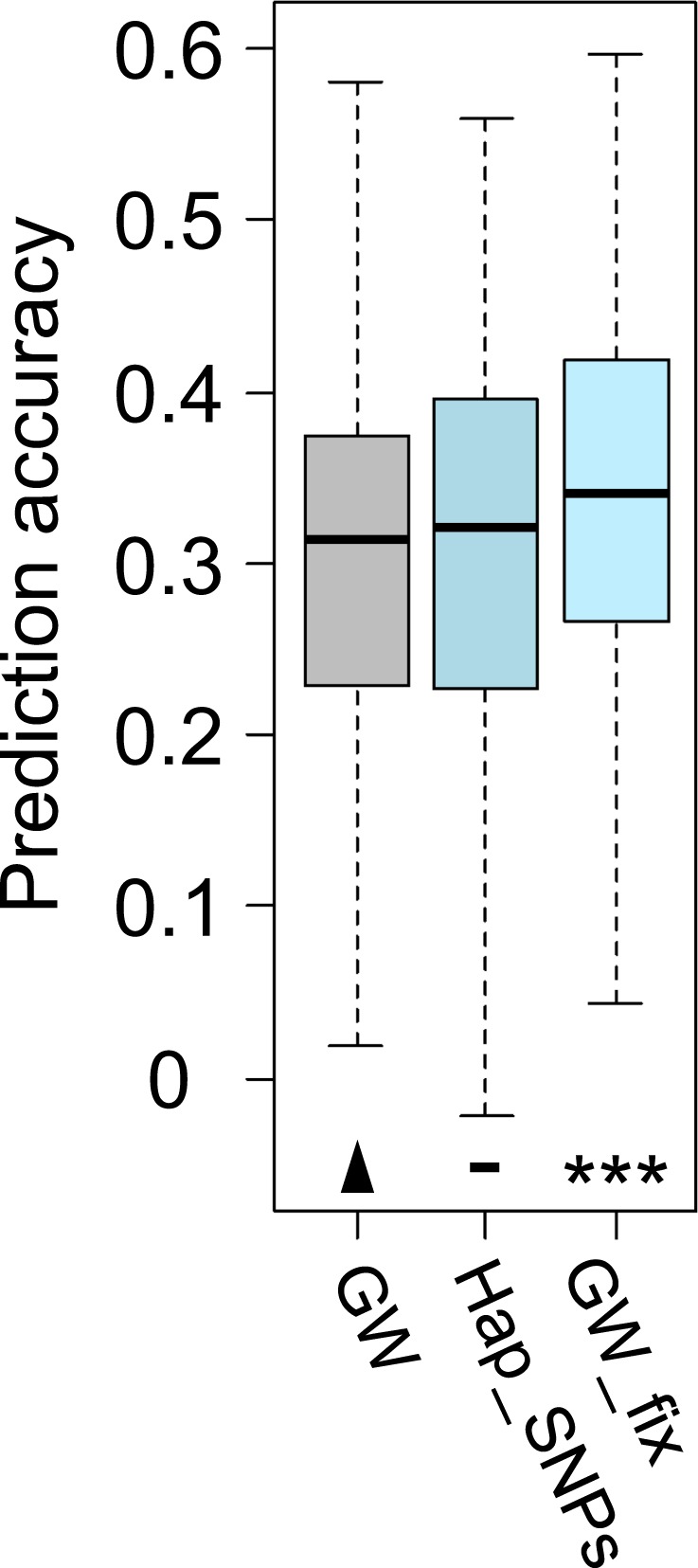
Comparison of prediction accuracies of three different genomic prediction models of the discoloration index. Model GW: All 18,885 polymorphic, genome-wide markers were included; Model Hap_SNPs: only the 31 defined haplotype markers were included; Model GW_fix: All 18,885 polymorphic, genome-wide markers were included and the six defined haplotypes were included as fixed effects in the model. Prediction accuracies measures as Pearson’s correlations between predicted and true phenotype values. Cross-validation with 500 runs was applied, where always 80% of the population was used as a training set to predict the phenotypes of the remaining 20%. A t-test was used to compare the prediction accuracies (p-values: − 0.1, *0.05 **0.01 ***0.001).

In order to evaluate the predictability of the discoloration score (the severity of stem-base lesions), we compared three different genomic selection (GS) models representing variations of the classical rrBLUP prediction model^22^. Overall, mean prediction accuracies were 0.305, 0.31 and 0.333 for predictions that included (a) all markers, (b) only the 31 haplotype-associated markers, and (c) all markers plus the six haplotype blocks modelled as fixed effects in the prediction model, respectively (Table S4). One-way ANOVA revealed a highly significant difference between the accuracies of predictions for the standard RR-BLUP model (using all genome-wide markers) and an extended model in which the six resistance-associated haplotypes were used as fixed effects in the model (p < 0.001, Fig. 3). Interestingly, there was no difference in accuracy between the genome-wide model and the one that only included the 31 significant GWAS markers (Table S5).

## Discussion

### Genome-wide association mapping reveals multiple small-effect loci and one major QTL

We used extensive RT-PCR experiments and discoloration scoring after artificial inoculation of greenhouse-grown wheat seedlings. RT-PCR-based approaches were previously also applied to investigate FRR and FCR caused by *F*. *culmorum*^23,24^ and *F*. *pseudograminearum*^23^. However, the focus in these studies was to investigate geographic occurrence of predominant pathogen species or physiological distribution of pathogen spread within the plant, but data generated from RT-PCR has not been used in genetic mapping yet. Although this procedure appears to provide a robust and relevant assessment of the plant resistance to FRR, the high cost and workload prohibit its general application in wheat breeding and research. On the other hand, our results demonstrate that parameters with low correlations with the trait of interest (i.e. discoloration score) can still be usefully incorporated in downstream data analyses for indirect application to breeding. A major effect QTL associated with FCR resistance caused by *F*. *pseudograminearum* and *F*. *graminearum* was previously mapped on the long arm of chromosome 3B bi-parental mapping populations^25–27^. This QTL, however, was not found in the present study. The population size in our study might limit the power to detect rare alleles, which can make up a significant fraction of the variance for a quantitative trait^28^. Nevertheless, the outcome of our GWAS for 21 resistance-related parameters suggests an absence of major-effect resistance genes against below-ground Fg infection with *F*. *graminearum*, instead implying a highly quantitative inheritance with a similar complexity to that described for FHB resistance^29^. For FHB caused by Fg, differences in floral anatomy (e.g. cell size and cell wall thickness) were correlated to resistance responses in wheat and barley^30^. Cell wall components like lignin can play a key role in disease response, as lignification processes enhance the plant defense against fungal attacks by establishing mechanical barriers and becoming more resistant to cell wall-degrading enzymes. Lignin was also shown to decrease the toxin diffusion and to suppress the nutrient supply of the pathogen^31^. Other studies also report that lignified cell walls are more resistant to cellulases and pectinases^32^ and that syringyl-rich lignin was accumulated in wheat cells during hypersensitive resistance responses^33^. It might be expected that similar anatomical characteristics of roots may also be involved in resistance to underground infection and spread of Fg, although investigation of such phenomena may be more challenging in root tissues than in aboveground plant organs. The negative correlations between higher biomass of roots and stems in control as well as in inoculated treatments with fungal DNA concentrations in roots also imply that genotypes with a higher total root biomass are more resistant to fungal infection through the roots. Increased total root biomass might be due to a higher amount of fine roots and/or a higher amount of roots with a different morphological composition on the cellular level in partially resistant genotypes, e.g. higher fiber/lignin content of root tissues resulting in a higher dry root biomass might lead to reduced susceptibility to infection. The latter is supported by the stronger correlation of root dry mass with the relative fungal DNA concentration (Fg_DNA_root_rel) than with the absolute DNA concentration (Fg_DNA_root_abs) in the roots. A potential explanation can also be that increased root growth represents a ‘tolerance reaction’ in which fungal infection is counteracted by an increased root production. Although root DNA concentrations are positively correlated with discoloration score and fungal infection significantly reduces plant biomass, the stem discoloration scores are weakly positively correlated with biomass, implying that visual stem discoloration scores are not suitable to judge FRR resistance, but may be biased by growth related discoloration of the stem base. Efficient breeding for FRR resistance thus will require development of cost-effective high-throughput DNA-based screening procedures for root phenotyping. The lack of correlation between the discoloration score at the stem base and the fungal DNA concentration in the stem implies that infection in the roots triggers symptom expression at the stem base and thus fungal root DNA concentrations, and not fungal DNA stem concentrations, should be used in resistance breeding for FRR.

### Has indirect selection accumulated FRR resistance-related alleles in European elite wheat varieties?

After grouping the genotypes in our diversity panel based on their number of resistance-associated haplotype alleles, we found that the discoloration score constantly decreased with an increasing number of resistance alleles. The relationship between an increased number of resistance factors and a decreased disease expression has been described for various crops and pathogens and is referred to as a “pyramiding effect”^34^. It has further been shown that resistance QTL can effectively enhance quantitative resistance against more than one disease^35^. For example, DNA markers have proven useful to combine multiple favorable QTL alleles, e. g. in rice where RFLP markers were used to accumulate resistance genes against bacterial blight^36^ or to improve fungal blast resistance^37^. In barley, pyramiding of resistance genes against the barley yellow mosaic virus complex resulted in a broader resistance spectrum against multiple virus strains^38^. In wheat, increased levels of powdery mildew resistance were achieved by a marker-based combination of different resistance genes^39^ and also for crown rot caused by *F. pseudograminearum*, SSR and DArT marker-based pyramiding of multiple QTL alleles was associated with a significantly lower disease severity. A further example is given by leaf rust resistance in wheat, a trait which is controlled by a large number of major gene- and quantitative resistance loci across the whole genome. It has recently been shown that there is a clear linear relationship between the total number of marker alleles associated with leaf rust resistance and the disease score^40^. In association with molecular genetic data, rapid generation advancement tools, such as speed breeding^41^, hold great potential for accelerated, marker-based accumulation of resistance alleles in order to achieve improved disease resistances in elite lines^42^.

A large number of QTL mapping studies have identified over 200 loci for resistance against FHB, caused by *F*. *graminearum*, in wheat. Despite this progress, the involvement of this pathogen in underground plant infections has remained largely unexplored. There are indications that plant responses to above- and below-ground infections of Fg have different underlying genetic mechanisms^10^. In a comparative study that investigated FHB and FCR resistance in a double haploid population, it was found that QTL for both resistances are located on different chromosomes, implying the involvement of different host genes in FHB and FCR resistance, respectively^10^. This also underlines the need for separate resistance screening systems for the two diseases. Screening for FRR presents specific challenges because root phenotyping in wheat is time-consuming, costly and difficult^16,43^. It is therefore highly unlikely that breeders have intentionally selected for resistance against FRR. Instead, it is more likely that resistance alleles have been inadvertently accumulated through indirect selection for improved plant performance in the field, resulting in unintentional allele stacking for FRR resistance in modern varieties. The observation that most genotypes combining five or six resistance haplotype alleles were from European wheat subpopulations, mainly consisting of high-yielding elite lines that were developed by intensive selective breeding, suggests that high disease pressure in European production environments may underlie this unintentional selection by European breeders. On the other hand, some lines from this group still showed an average stembase discoloration score of 3 out of maximal 5 highlights the need for further work in order to increase resistance against underground plant infection with Fg.

### Resistance-associated haplotypes improve genomic prediction accuracies

Recent advances in next-generation sequencing technologies and the availability of commercial high-throughput genotyping platforms make it feasible to generate detailed genome-wide genotype profiles, even for crops with complex polyploid genomes, at constantly decreasing costs^44^. In conjunction with novel statistical solutions to exploit genomic data, these techniques have revolutionised wheat breeding research during the past decade^45^. Genomic selection (GS), a form of marker-assisted selection which considers genome-wide marker data to predict the performance of a genotype based on genomic estimated breeding values, is being increasingly used in plant breeding for selection of complex traits. The major advantage of GS in plants is the ability to (pre-)select candidate genotypes as seeds or seedlings, rather than after years of intensive, expensive phenotyping. This enables breeders to reduce the duration of the breeding cycle and ultimately increase the rate of genetic gain per unit of time^46–48^. A number of studies have described the successful application of GS in resistance breeding approaches, including FHB^49–51^ and stem rust resistance^52^. For soil-borne diseases, GS would be of particular importance, as phenotyping in this case is particularly difficult, time-consuming and costly. In recent FHB studies it was shown that GS clearly outperformed marker-assisted selection, implying that GS can be a powerful tool to efficiently improve FHB resistance^50,53^.

Initially, applying three basic RR-BLUP models, we obtained relatively low prediction accuracies. This can partly be explained by the fact that the training population in our cross-validations was too small to sufficiently capture important rare alleles for a reliable prediction of marker allele effects. Furthermore, our population included genotypes from genetically very distant eco-geographic germplasm pools, e.g. lines from China vs. lines from Europe, which strongly reduced the relatedness between the training and prediction population in most of the cross-validation runs. This is one main factor negatively impacting prediction accuracy in GS^54^. On the other hand, it has previously been shown that markers identified in previous GWAS runs could improve the predictability for different traits in rice when modeled as fixed effects in the prediction model^55^. In another study, we suggested an extension of this approach by using haplotypes, the combination of multiple markers that are associated with the trait of interest, to increase the resolution at which complex relationships between quantitative phenotypes and molecular variations can be depicted^28^. Here, modelling the previously defined resistance haplotypes as fixed effects in the prediction model resulted in significantly improved prediction accuracies. Even though the absolute effect was only moderate, the fact that predefined haplotypes could significantly improve the accuracy even under very unfavorable GS conditions (small, very diverse training population) highlights the huge potential of this approach to improve GS designs. We also demonstrate the power of RT-PCR to provide reliable and consistent phenotypic values for a complex, underground disease that breeders otherwise have extreme difficulties to assay. We expect that the use of PT-PCR to train GS models could significantly advance breeding progress for FRR in wheat.

## Methods

### Plant material and genome-wide SNP marker data

A diverse collection of 215 homozygous wheat lines, representing species-wide diversity from China, Europe, North America and Australia, was selected from a global panel of 460 bread wheat varieties^16,56^ based on population genetic analysis. The panel was genotyped with the 90,000-SNP Illumina Infinium wheat genotyping array (Illumina Inc., San Diego, CA, USA)^21^. The raw genotype data was filtered to omit markers with ≥10% missing values and minor allele frequency ≤5%, resulting in 18,855 high-quality, polymorphic SNPs for the subsequent genetic analysis. All SNP markers used for subsequent analyses were ordered according to their genetic positions in a high-resolution consensus map^21^.

### Infection experiment and phenotypic FRR resistance assessment

Plant and pathogen cultivation was performed following Wang *et al*.^20^. Briefly, plants of 215 wheat lines were grown in greenhouse experiments (21/15 C day/night, 16 h photoperiod), in three replicates of five plants each, between December 2014 and March 2015. An augmented block design with nine sub-experimental blocks was applied in which 16 to 26 wheat lines from the diversity panel plus four control wheat lines with previously evaluated varying reactions to fungal infection (data not shown) were evaluated in each sub-experiment. Adjusted entry means were calculated for each line and each trait using a mixed linear model that considered “genotypes”, “infection treatment” (control or infected) and their interaction as fixed, and the sub-experimental block effect as random effects. Seeds were sterilized in 6% sodium hypochlorite and sown in autoclaved soil-sand mixture (1:2 vol/vol). Seven days after sowing, plants were removed from the growth medium and transferred into a small flat tray by submerging their roots in 5 ml of *F*. *graminearum* macroconidia suspension with a concentration of 50,000 spores/ml. Seedlings were held in place by a slot in a styrofoam plug and were kept on a rotary shaker for 2 h. Mock plants that represent the uninfected control were placed in a deionized water bath for 2 h. Subsequently, plants were transferred into sterilized 12 cm diameter plastic pots filled with autoclaved sand. Plants were continuously irrigated with 0.2% WUXAL® Super NPK hydroponic fertilizer (Wilhelm Haug GmbH & Co. KG, Düsseldorf, Germany). Thirty-five days after sowing plants were removed from the pots and roots were carefully washed. After measuring of shoot and root length, from the base of the seedling coleoptile to the respective outermost extremity, high resolution image recording for subsequent visual discoloration scoring was performed using a Canon EOS5 camera. As disease symptoms on roots were not evident, the scoring was only performed for the stem-bases of the plants. Discoloration scoring was done for the inoculated plants only, using a 1–5 scale for each individual plant, where 1 represents symptomless stem-bases and 5 means a total necrosis. The discoloration score per replicate was calculated as

$${\rm{Discoloration}}\,{\rm{score}}=\frac{1\ast {\rm{X}}1+2\ast {\rm{X}}2+3\ast {\rm{X}}3+4\ast {\rm{X}}4+5\ast {\rm{X}}5}{{No}.{plants}\,{per}\,{replicate}}$$

where ${\rm{X}}1\mbox{--}{\rm{X}}5\,\,$represent the number of plants with symptom scores between 1 and 5, respectively. In order to increase the reliability, photographs were evaluated in three independent runs and an overall mean was calculated for each line (Table S6). Immediately after harvesting, plants were freeze-dried at −60 °C for 7 d before measurement of total root and shoot dry biomass for each accession and tissue sampling for subsequent DNA extraction.

### Heritability estimation

Except for the four check lines which were replicated across all nine sub-experimental blocks, each individual genotype from the diversity collection was only evaluated in one of the nine sub-experiments, so the overall genotype x environment interaction effect could not be estimated across all experiments as it was confounded with the statistical residual term. Therefore, we used the following approximation method for heritability estimation in unbalanced data as described by Piepho and Möhring^57^,

$${{H}}^{2}=\frac{{\sigma }_{G}^{2}}{{\sigma }_{G}^{2}+\frac{1}{2}\overline{vd}}$$

where *H*^2^ represents the broad-sense heritability, ${\sigma }_{G}^{2}$ represents the genotypic variance calculated with a random effect model and $\overline{vd}$ represents the average variance of a difference between two line means, obtained by squaring the standard error of the difference between two line means. To calculate the latter parameter, we used the R package “lsmeans”^58^.

### Real-time PCR analysis

Genomic DNA from wheat and fungus tissues was isolated using the same method described by^20^. Analyses were performed on a ViiA 7 Real-time PCR system (Applied Biosystems, Foster City, CA, U.S.A.). For instrument calibration we used a six-step dilution series of 30 (10), 15 (2), 7.5 (0.4), 1.875 (0.1), 0.469 (0.01) and 0.117 (0.001) ng per microliter for plant and fungal tissue, respectively. The following primers were used: *F*. *graminearum*–specific fragment 16N-F (ACAGATGACAAGATTCAGGCACA) and 16N-R (TTCTTTGACATCTGTTCAACCCA) and the wheat Ubiquitin gene (DQ086482/Ta.28553.1.S1_s_at) with primers Ubi-F (CCCTGGAGGTGGAGTCATCTGA) and Ubi-R (GCGGCCATCCTCAAGCTGCTTA)^20^. The final amplification mix consisted of 1 μl of template DNA, 5 μl of Roche FastStart SYBR green master (Roche Diagnostics GmbH, Mannheim, Germany), 2 μl of double-distilled water, 1 μl forward and 1 μl reverse primer (10 pmol/μl each). PCR amplification was carried out using an initial denaturation step for 3 min at 94 °C, which is followed by 36 reaction cycles consisting of a 15 s denaturation step at 94 °C, an annealing step for 20 s at 57 °C and 40 s at 72 °C. The final elongation was performed for 5 min at 72 °C. During the amplification process, the detection of fluorescence was carried out in the annealing step of each cycle. To verify amplification of the specific target DNA, a melting curve analysis was included. Melting curves were acquired by heating the samples to 95 °C for 1 min, cooling to 55 °C for another min and then slowly increasing the temperature from 65 to 95 °C at the rate of 0.5 °C s^−1^, with a continuous measurement of the fluorescence. Gene expression values were normalized to the housekeeping gene *Ubiquitin* values using a 2^−ΔCt^ method^59^. The method was modified by including the PCR efficiency in the normalization process, namely E^−ΔCt^ where ΔCt = (Ct^fg16^ − Ct^ubiquitin^) and E = 10^−1/slope^. RT-PCR analyses resulted in fold-change values of the fungus-specific gene fragment 16 N relative to *Ubiquitin* in roots and stembases, designated as the parameters Fg_DNA_root_fc and Fg_DNA_stem_fc, respectively. We also investigated the absolute amount of fungal DNA in the total of 15 ng DNA per sample, designated Fg_DNA_root_abs and Fg_DNA_stem_abs. To account for anatomical differences between genotypes, like differences in cell size or thickness of cell walls we also calculated a relative value for fungal DNA in roots by dividing the absolute amount in 15 ng DNA by the absolute root dry mass, designated Fg_DNA_roots_rel.

### Genome wide association analysis, haplotype construction and genomic prediction

Genome-wide marker-trait associations were calculated from adjusted entry means for each genotype (Table S1), using the R package GenABEL^60^ and a two-step mixed linear model approach that increases detection power without increasing the empirical type I error^61^. The model was adjusted for population stratification by including identity-by-state estimates for genotype pairs and a principal component adjustment that uses the first two principal components as covariates. For identification of significant marker-trait associations, a relaxed significance cutoff value was set at −log_10_(p-value) = 3 in order to reduce the type II error rate. Based on analysis of local LD surrounding markers with significant associations (r^2^ > 0.7) to RT-PCR scores, we constructed one major haplotype for each of the traits Fg_DNA_stem_abs/-fc, Fg_DNA_root_abs/-fc/-rel and the discoloration score, respectively. For each of the six identified haplotypes, genotypes were grouped according to their allelic haplotype state and phenotypes were compared between groups. Haplotype alleles that were associated with a resistance-related parameter score (e.g. lower total fungal DNA in root tissue) were designated “resistance alleles”. To investigate an effect of accumulated resistance alleles at the independent haplotype blocks on the discoloration score we assigned the lines from the diversity panel to six groups, based on the absolute number of resistance alleles they carried (1–6). We then compared discoloration scores between those six groups. In order to evaluate the predictability of the discoloration score using SNP markers we compared three linear statistical genomic selection models, basically representing three variations of a ridge regression BLUP^22^. In the first model (*i-GS_wg*), all genome-wide markers were included as random effects, in the second model (*ii-GS_hap*) only the identified 31 haplotype-related markers were used as random effects for predictions and in the third model (*iii-GS_wg* + *fixhap*) fixed effects for the six identified haplotypes, related to fungal DNA content and discoloration score were included. The general form of all statistical models is represented by the following equation

$${y}={X}{\rm{\beta }}+{Zu}+{e}$$

in which $y$ is a *n* × 1 vector of phenotypic adjusted entry means and n the number of wheat lines. *X* is an incidence matrix relating fixed effects to individuals, and ${\rm{\beta }}$ is the corresponding vector containing the respective effects. In the cases if the first two models, *X* only assigns the overall phenotypic mean μ to all lines (then $X{\rm{\beta }}$ simply can be replaced by $\,{1}_{n}\mu$), but haplotypes defined as fixed factors can also be included (cv. *iii-GS_wg* + *fixhap*). *Z* is a design matrix relating the allele calls of all SNPs to the individuals, *u* is a vector of random SNP effects and *e* represents the residual error.

## Electronic supplementary material

Supplementary Information

Supplementary Tables
