# Supplementary material for: Genetic insights into underground responses to Fusarium graminearum infection in wheat
Source: Sci Rep. 2018 Sep 3;8:13153. doi: 10.1038/s41598-018-31544-w (PMC6120866; doi:10.1038/s41598-018-31544-w)
Supplement: Supplementary file 1 — Supplementary Information [file 41598_2018_31544_MOESM1_ESM.docx]

**Supplementary Information for**

**Genetic insights into underground responses to *Fusarium graminearum* infection in wheat**

Kai P. Voss-Fels^1,2^*^#^, Lunwen Qian^1,3^*, Iulian Gabur^1^, Christian Obermeier^1^, Lee T. Hickey^2^, Christian R. Werner^1^, Stefan Kontowski^4^, Matthias Frisch^5^, Wolfgang Friedt^1^, Rod J. Snowdon^1^, Sven Gottwald^1^

^1^ Department of Plant Breeding, IFZ Research Centre for Biosystems, Land Use and Nutrition, Justus Liebig University, Heinrich-Buff-Ring 26-32, 35392 Giessen, Germany

^2^ Queensland Alliance for Agriculture and Food Innovation, The University of Queensland, St Lucia, QLD, 4072, Australia

^3^ Collaborative Innovation Center of Grain and Oil Crops in South China, Hunan Agricultural University, Changsha 410128 P.R. China

^4^ W. von Borries-Eckendorf GmbH & Co. KG, Hovedisser Str. 92, 33818 Leopoldshöhe, Germany

^5^ Institute for Agronomy and Plant Breeding II, IFZ Research Centre for Biosystems, Land Use and Nutrition, Justus Liebig University, Heinrich-Buff-Ring 26-32, 35392 Giessen, Germany

* Joint first authors

^#^ Corresponding author:

Kai Voss-Fels, Queensland Alliance for Agriculture and Food Innovation, The University of Queensland, St Lucia, QLD, 4072, Australia

Email: k.vossfels@uq.edu.au

Phone: +61 7 334 62288

Fax: +61 7 334 60555

**This file includes:**

**Supplementary Figures:** Figure S1-S4

**Supplementary Table legends:** Table S1-S6 (provided in separate excel file)

**
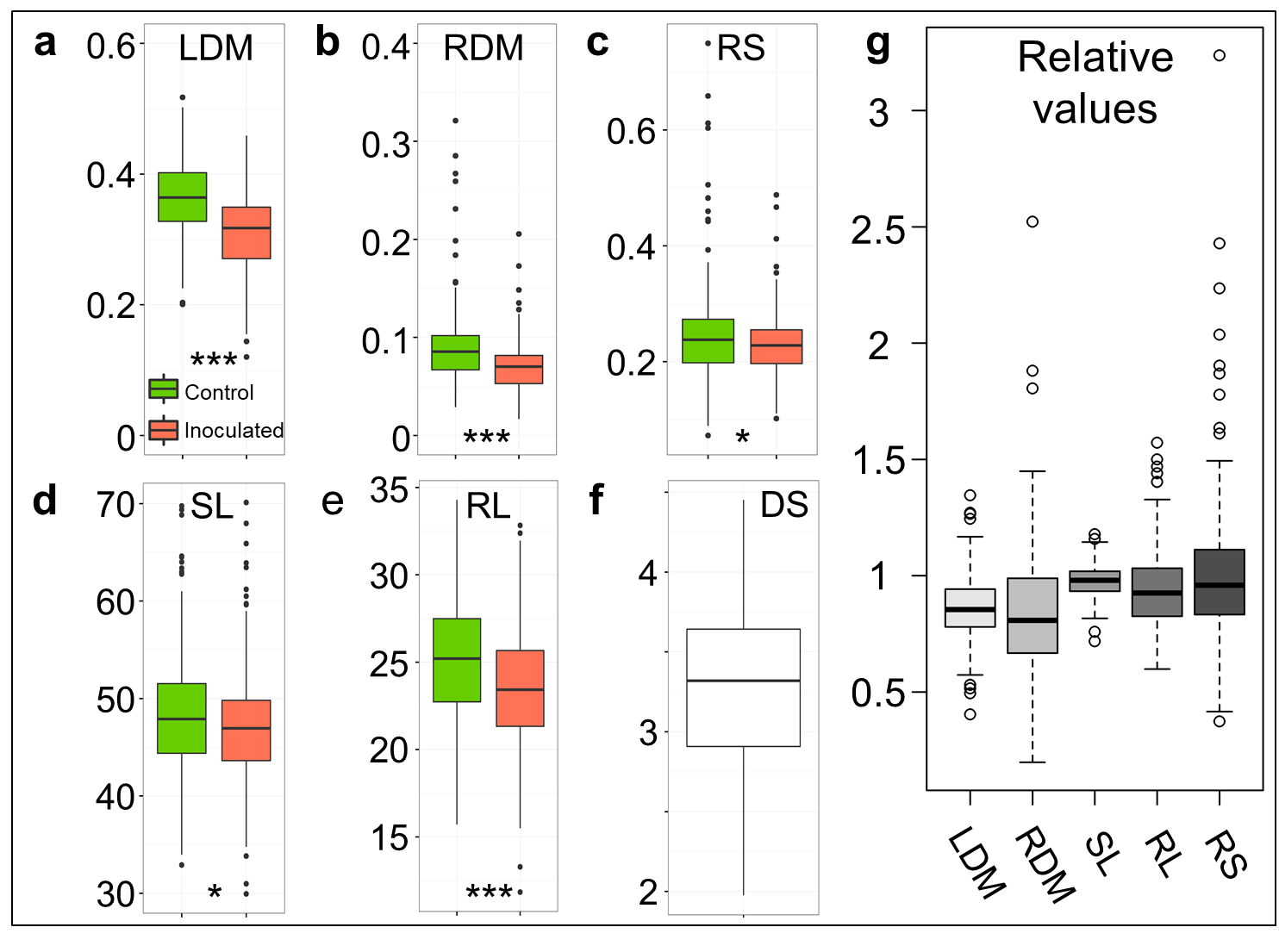
**

**Figure S1** Comparison of basic seedling traits under controlled and infected conditions. (a)-(e) Stars indicate p-values from an ANOVA comparing the phenotype data of the two groups: * = 0.05, ** = 0.01 and *** = 0.001. LDM= leaf dry matter; RDM= root dry matter; SL= shoot length; RL= root length; RS= root-to-shoot ratio; (f) DS= discoloration score; (g) Relative values calculated as ‘infected’/’control’.


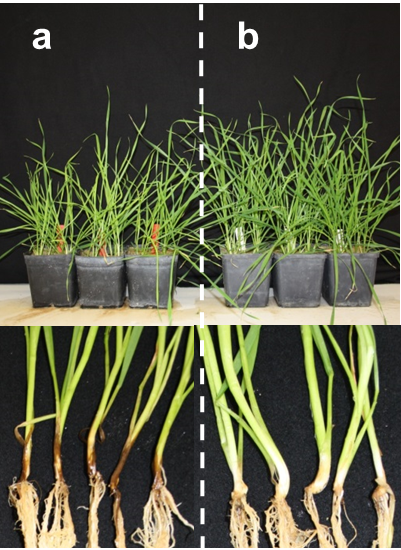


**Figure S2** Phenotypic effects of *F. graminearum* inoculation in 35 days old wheat plants. Wheat line 418 is shown (a) after inoculation with *Fg* (mean discoloration score = 3.9) and (b) in the control version, inoculated with water. The ratio of leaf dry mass under infected to controlled conditions (rLDM) is 0.67.


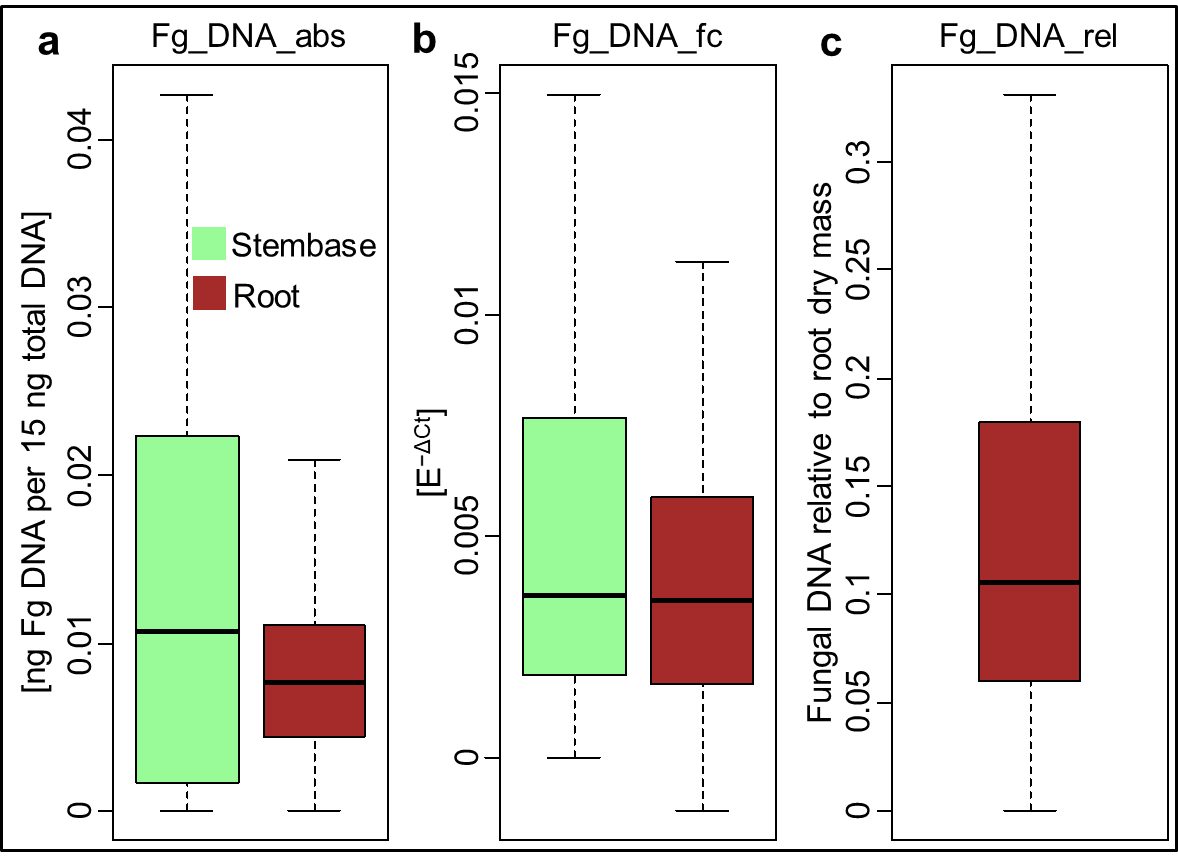


**Figure S3** Summary of data for three different RealTime-PCR parameters. Fg= *Fusarium graminearum*; (a) abs= absolute amount of fungal DNA; (b) fc= fold-change of *F. graminearum*-specific gene related to *Ubiquitin*; (c) rel= absolute fungal DNA amount divided by total root dry mass under infection.


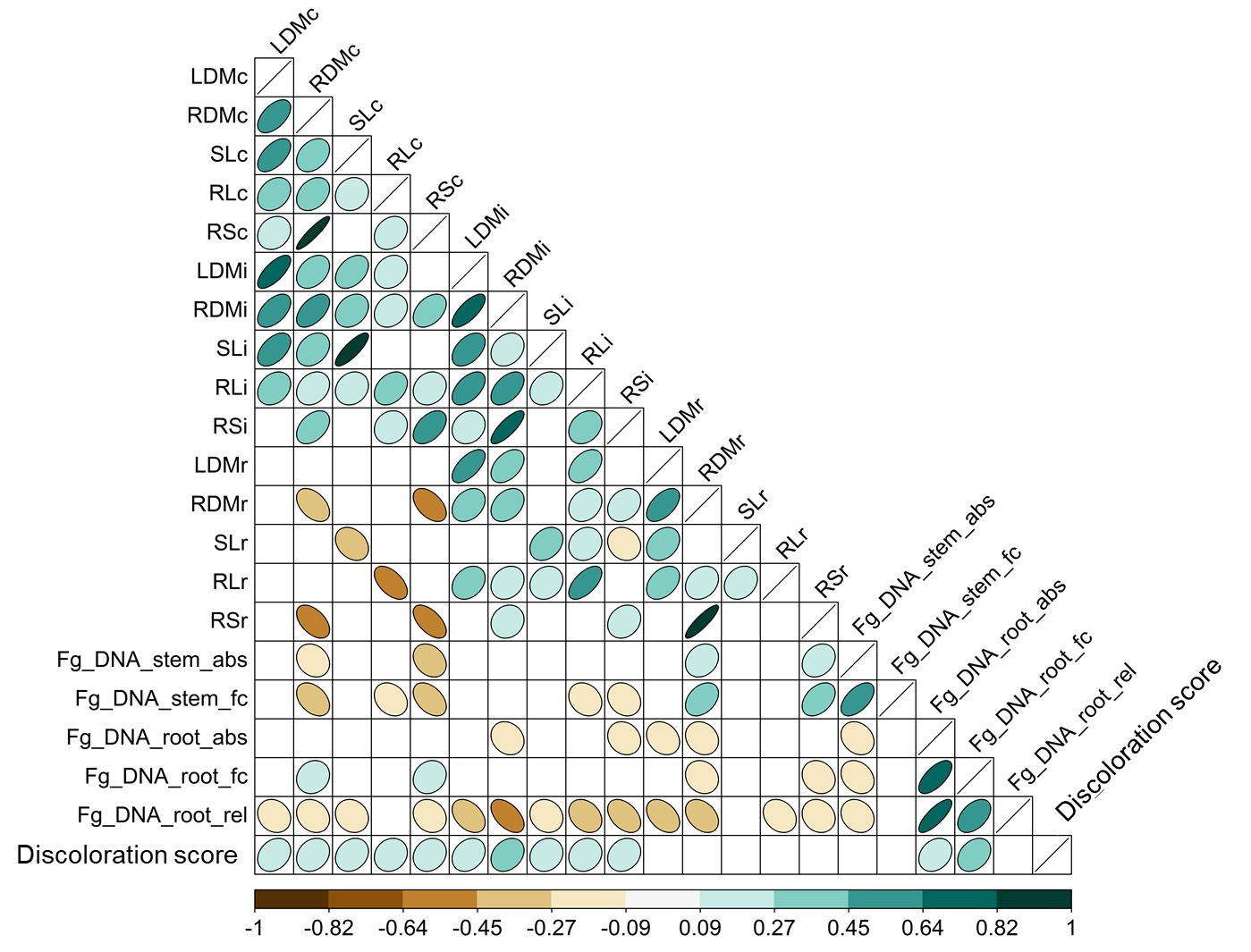


**Figure S4** Full correlation matrix for all 21 traits measured in 215 wheat lines. Correlations were measured as Pearson’s correlation coefficient. Only significant correlations shown (p < 0.05). LDM= leaf dry matter; RDM= root dry matter; SL= shoot length; RL= root length; RS= root-to-shoot ratio; c= control; i= infected; r= relative value (between i and c); Fg= *Fusarium graminearum*; abs= absolute amount of fungal DNA in measured sample; fc= fold-change of Fg-specific gene related to *ubiquitin*; rel= abs / RDMi.

**Supporting Tables summarized in a separate excel file**

**Table S1.** Genotype list of the tested wheat lines.

**Table S2.** Adjusted mean values for all 21 traits.

**Table S3.** Genome-wide association mapping summary for marker-trait associations with a -log10(p-value) > 3.

**Table S4.** Prediction accuracies of three different genomic prediction models from 500-fold cross validations with 80% training and 20% prediction population for each run.

**Table S5.** List of haplotype markers.

**Table S6.** Raw data of three independent discoloration scoring runs.
